# Association between serum uric acid and triglycerides in Chinese children and adolescents with short stature

**DOI:** 10.1186/s12944-020-01429-x

**Published:** 2021-01-06

**Authors:** Yuntian Chu, Qianqian Zhao, Mei Zhang, Bo Ban, Hongbing Tao

**Affiliations:** 1 School of Medicine and Health Management, Tongji Medical College, Huazhong University of Science and Technology, 13 Hangkong Road, Qiaokou district, Wuhan, 430030 Hubei China; 2 Department of Endocrinology, Affiliated Hospital of Jining Medical University, Jining Medical University, 89 Guhuai Road, Rencheng District, Jining, 272029 Shandong China; 3 Chinese Research Center for Behavior Medicine in Growth and Development, Jining, 272029 Shandong China

**Keywords:** Serum uric acid, Triglycerides, Body mass index, Cardiovascular disease, Short stature, Children and adolescents, Non-linear relationship

## Abstract

**Background:**

Elevated triglyceride (TG) levels are a biomarker for cardiovascular disease (CVD) risk. The correlation between serum uric acid (SUA) and TG concentrations in adults or obese children is well established. However, studies on SUA and TG in children with short stature are limited.

**Aim:**

To determine the relationship between SUA and TG levels in short children and adolescents.

**Method:**

This was a cross-sectional evaluation of a cohort of 1095 patients with short stature (720 males and 375 females). The related clinical characteristics, including anthropometric and biochemical parameters, were determined.

**Results:**

Smooth curve fitting, adjusted for potential confounders was performed, which indicated the existence of a non-linear relationship between these measures. Piecewise multivariate linear analysis revealed a significant positive relationship between SUA and TG at SUA concentrations over 7 mg/dL (β = 0.13, 95% CI: 0.05–0.22, *P* = 0.002) but no significant correlation at lower SUA levels (β = 0.01, 95% CI: 0.01–0.04, *P* = 0.799). Furthermore, a stratified analysis was performed to appraise changes in this relationship for different sexes and standard deviation levels of body mass index (BMI). The non-linear relationship remained consistent in males and females with BMI standard deviation scores (BMI SDS) ≥ 0, with inflection points of 6.71 mg/dL and 3.93 mg/dL, respectively. Within these two groups, SUA and TG levels showed a positive association when SUA levels were higher than the inflection point (β = 0.21, 95% CI: 0.11–0.31, *P* < 0.001 for males and β = 0.1, 95% CI: 0.03–0.17, *P* = 0.005 for females). However, a specific relationship was not observed at lower SUA levels. No significant relationships were found between SUA and TG levels in males and females with BMI SDS < 0.

**Conclusion:**

The present study identified the non-linear association of SUA and TG levels with short children and adolescents. This relationship was based on BMI status. This finding suggests that health status should be considered for short stature children with high SUA levels, especially in children with a high BMI standard deviation score.

**Supplementary Information:**

The online version contains supplementary material available at 10.1186/s12944-020-01429-x.

## Background

One of the most common reasons for referral to a growth and development specialist is short stature. The factors affecting stature are complex and diverse—they include nutrition, adjusting hormonal conditions, pubertal stage, and chronic conditions occurring during the foetal to adolescent period. Children and adolescents with short stature not only have height problems but also experience psychological and physiological effects [1, 2]. Findings in previous prospective studies and meta-analyses showed the probability of an existing negative correlation between height and cardiovascular disease (CVD) risk [3–6]. This probability of a negative association also exists between height and lipid levels for both children and adults [7–9]. Hence, it is necessary to pay attention to the lipid profile of short children as a risk factor for CVD. Lipid levels in adulthood can usually be traced back to childhood levels. Although CVD is not very common in young people, the accumulation of intimal fat streaks is an early form of atherosclerosis that accompanies dyslipidaemia in childhood [10–12].

Dyslipidaemia is an established risk factor for CVD, especially abnormal levels of low-density lipoprotein cholesterol (LDL-C) and apolipoprotein B [13, 14]. However, the number of studies showing elevated triglyceride (TG) levels as a biomarker for CVD are increasing [15–17]. Evidence shows that elevated residual cholesterol, marked by elevated TG levels, is another increasingly identified prevalent causal risk factor for CVD [18]. A meta-analysis involving 10,158 patients with CVD among 29 Western prospective studies showed that TG levels were highly associated with CVD [19]. Serum uric acid (SUA) is the final metabolite of purines and is excreted mainly by the kidneys and intestines. Previous studies have shown that SUA levels reflect a strong risk factor for CVD [20–24]; furthermore, an association between SUA levels and CVD mortality exists [25, 26]. Either reduced excretion or enhanced synthesis of SUA could give rise to abnormal elevations in its concentration, while the process of synthesising fatty acid (i.e., TG) that occurs in the liver is related to de novo synthesis of purines that accelerate the production of SUA [27]. Several early studies showed that SUA levels are connected with TG levels in adults and obese children [21, 28–30]. However, studies on SUA and TG in short stature children who may have a greater risk of developing cardiovascular conditions in adulthood have been limited. In addition, insulin-like growth factor 1 (IGF-1) is another possible link contributing to the correlation of the two indicators, as IGF-1 is related to lipid profiles in short children [14] and with SUA levels [31]. Therefore, the present study examines the relationship between SUA and TG in short children and adolescents.

## Methods

### Subjects

The study group consisted of 1095 participants with short stature (720 males and 375 females) who visited the Endocrinology Department at the Affiliated Hospital of Jining Medical University between March 2013 and March 2020. A cross-sectional evaluation in the cohort of short stature population was performed, with a median age of 10.9 years. Short stature was defined as a condition for an individual who was 2 or more standard deviation scores shorter than the mean height for the population in given age, sex, or race [32]. Subjects with a normal birth weight and birth length and without chronic disease were included in the study. Among them, subjects with precocious puberty, congenital adrenal hyperplasia, chondrodysplasia, and genetic or chromosomal abnormalities (e.g., Turner syndrome) were excluded.

### Anthropometric measurements

Each study subject’s height and weight were measured by an individual at the growth and development specialist clinic. Subjects were measured in the morning, wearing casual clothes and no shoes, using standard methods. Height was assessed using a height measuring apparatus (Nantong Best Industrial Co, Ltd., Jiangsu; China), which has a maximum valid error of 0.1 cm. Weight was measured using a scale (Wuxi Weigher Factory Co., Ltd., Jiangsu, China) with a precision of 0.1 kg. Height SDS were generated from the standard values of Chinese children [33]. Body mass index (BMI) was calculated as weight in kilograms divided by the square of height in metres. BMI SDS values were calculated in accordance with development figures for Chinese children and teenagers published in 2009 [34]. The puberty stage was appraised based on physical examination and the Tanner stage [35]. Boys without pubic hair who also had a testicular size under four mL and girls without pubic hair who had undeveloped breasts were considered prepubertal.

### Laboratory measurements

Laboratory parameters were measured from fasting blood samples collected from all participants. A biochemical automatic analyser was used to detect the following indexes: blood lipids—TG, total cholesterol (TC), high-density lipoprotein cholesterol (HDL-C) and LDL-C and renal function—creatinine (Cr), urea nitrogen (BUN) and SUA (Cobas c702, Roche; Shanghai, China). A chemiluminescence assay with intra-assay and inter-assay parameters with variations of 3.0 and 6.2%, respectively (DPC IMMULITE 1000 analyser, SIEMENS, Berlin, Germany), was performed to detect serum IGF-1 levels. IGF-1 SDS got counted by IGF-1 degrees derived from age- and sex-matched robust children and teenagers [36].

### Statistical analysis

Continuous variables are displayed as the median (interquartile range). Categorical variables are shown as numbers and percentages. The differences in biochemical and clinical features for male and female participants were estimated by Kruskal-Wallis tests for constant variants and chi-square tests for categorical variants. To determine the factors affecting TG levels, a univariate analysis was performed. General additive models, adjusting for underlying confounders, were applied to obtain a smoothing curve and identify any non-linear association between SUA and TG levels. A variance inflation factor analysis was performed for every confounder in the multivariable regressions to test for potential multicollinearity problems. Adjusted R squared values were calculated to assess goodness of fit for each model, and log-likelihood ratio tests were applied to compare piecewise models and linear models adjusted for the same confounders. Two-sided *P* values under 0.05 for hypothesis tests were regarded as statistically significant. All analyses were conducted with EmpowerStats Software (http://www.empowerstats.com, X&Y Solutions, Inc., Boston, MA), coding in R version 3.6.1 (http://www.r-project.org).

## Results

### Clinical and biochemical characteristics of the subjects

The clinical and biochemical features of all subjects are summarized in Table 1. The distribution of the continuous variables was tested, and none fit the normal distribution (Table S1). A total of 1095 subjects with a median age of 10.9 years were analysed in this study, of which 65.75% were male and 56.99% (624) were prepubescent. The subjects’ median height SDS was − 2.57 (− 3.13--2.25), with no significant difference between the values of − 2.59 (− 3.18--2.26) for males and − 2.52 (− 3.07--2.23) for females (*P* = 0.205). The median TG and SUA levels were 0.69 (0.53–0.91) mmol/L and 4.52 (3.78–5.50) mg/dL, respectively. There was no difference in TG levels between males and females (*P* = 0.201), and SUA concentration distribution was significantly greater in males than in females (*P* = 0.010).

**Table 1:** Clinical and biochemical characteristics

	All	Male	Female	*P*
Number	1095	720 (65.75%)	375 (34.25%)	–
Age (years)	10.9 (8.4–13.1)	11.8 (8.4–13.5)	9.8 (8.2–11.6)	< 0.001
Height (cm)	134.20 (119.70–144.40)	137.20 (118.97–147.43)	131.50 (120.85–139.80)	< 0.001
Height SDS	−2.57 (−3.13--2.25)	− 2.59 (− 3.18--2.26)	−2.52 (− 3.07--2.23)	0.205
Body weight (kg)	30.00 (22.00–39.00)	31.00 (22.00–41.00)	29.00 (22.00–35.00)	< 0.001
BMI (kg/m^2^)	16.67 (15.16–19.01)	16.66 (15.18–19.37)	16.67 (15.13–18.56)	0.275
BMI SDS	− 0.17 (− 0.92–0.75)	− 0.21 (− 0.92–0.71)	−0.08 (− 0.95–0.80)	0.512
IGF-1 (ng/mL)	198.00 (114.75–321.00)	186.50 (108.00–295.75)	220.00 (138.50–359.00)	< 0.001
IGF-1 SDS	− 1.02 (− 1.84--0.18)	−1.00 (− 1.84--0.15)	−1.10 (− 1.84--0.20)	0.803
TG (mmol/L)	0.69 (0.53–0.91)	0.68 (0.53–0.90)	0.70 (0.54–0.94)	0.201
TC (mmol/L)	3.78 (3.37–4.25)	3.77 (3.37–4.24)	3.79 (3.38–4.26)	0.703
HDL-C (mmol/L)	1.33 (1.18–1.54)	1.34 (1.18–1.54)	1.33 (1.18–1.54)	0.727
LDL-C (mmol/L)	2.03 (1.70–2.39)	2.00 (1.70–2.36)	2.08 (1.74–2.40)	0.230
SUA (mg/dL)	4.52 (3.78–5.50)	4.60 (3.80–5.65)	4.40 (3.75–5.26)	0.010
Cr (μmol/L)	40.10 (34.02–46.40)	41.40 (34.82–48.50)	37.80 (32.60–43.12)	< 0.001
BUN (mmol/L)	4.40 (3.70–5.30)	4.70 (3.90–5.50)	4.10 (3.50–4.70)	< 0.001
Pubertal stage				< 0.001
In prepuberty (%)	624 (56.99%)	451 (62.64%)	173 (46.13%)	
In puberty (%)	471 (43.01%)	269 (37.36%)	202 (53.87%)	

Abbreviations: *Height SDS* Height standard deviation scores, *BMI SDS* Body mass index standard deviation scores, *IGF-1 SDS* Insulin like growth factor-1 standard deviation scores, *TG* Triglyceride, *TC* Total cholesterol, *HDL-C* High density lipoprotein-cholesterol, *LDL-C* Low density lipoprotein cholesterol, *SUA* Serum uric acid, *BUN* Blood urea nitrogen, *Cr* Creatinine. Data are presented as median (interquartile range) or percentage. *P* < 0.05 is considered to be statistically significant

### Factors associated with TG levels

The correlations between TG and SUA levels and other anthropometric and biochemical variables in the univariate linear regression analysis are displayed in Table 2. SUA and TG levels were significantly positively associated (*P* < 0.001) in males but not in females. In addition, TG levels had a positive relationship to weight and TC and LDL-C levels in both sexes (*P* < 0.05). In contrast, one significant negative relationship was found between HDL-C and TG levels (*P* < 0.001). None of the remaining relationships, such as those between TG and height SDS, BMI SDS, IGF-1 SDS or Cr, were significant in males or females (all *P* > 0.05).

**Table 2:** Association between TG levels and different variables by univariate analysis

Variables	All		Male		Female	
	β (95% CI)	*P* value	β (95% CI)	*P* value	β (95% CI)	*P* value
Age (years)	0.01 (0.01, 0.02)	< 0.001	0.01 (0.01, 0.02)	< 0.001	0.01 (− 0.01, 0.02)	0.133
Height SDS	−0.01 (− 0.04, 0.02)	0.462	−0.01 (− 0.04, 0.04)	0.964	−0.03 (− 0.07, 0.01)	0.134
Body weight (kg)	0.008 (0.006, 0.010)	< 0.001	0.008 (0.006, 0.010)	< 0.001	0.009 (0.006, 0.013)	< 0.001
BMI SDS	−0.01 (− 0.02, 0.02)	0.853	−0.01 (− 0.04, 0.01)	0.344	0.02 (− 0.01, 0.05)	0.071
IGF-1 SDS	−0.01(− 0.03, 0.01)	0.854	−0.01(− 0.03, 0.02)	0.865	−0.02 (− 0.05, 0.01)	0.176
TC (mmol/L)	0.06 (0.03, 0.10)	< 0.001	0.05 (0.01, 0.10)	0.033	0.08 (0.03, 0.13)	< 0.001
HDL-C (mmol/L)	−0.35 (− 0.43, − 0.27)	< 0.001	−0.42 (− 0.53, − 0.32)	< 0.001	−0.23 (− 0.35, − 0.11)	< 0.001
LDL-C (mmol/L)	0.11 (0.07, 0.15)	< 0.001	0.12 (0.07, 0.18)	< 0.001	0.09 (0.03, 0.15)	0.005
SUA (mg/dL)	0.05 (0.04, 0.07)	< 0.001	0.06 (0.04, 0.09)	< 0.001	0.03 (0.01, 0.06)	0.359
Cr (μmol/L)	0.01 (− 0.01, 0.01)	0.388	0.01 (− 0.01, 0.01)	0.268	0.01 (− 0.01, 0.01)	0.947
BUN (mmol/L)	−0.01 (− 0.01, 0.01)	0.132	−0.01 (− 0.01, 0.01)	0.316	−0.05 (− 0.09, − 0.01)	0.007
Pubertal stage						
In prepuberty (%)	reference		reference		reference	
In puberty (%)	0.06 (0.02, 0.11)	0.009	0.05 (−0.01, 0.11)	0.125	0.09 (0.02, 0.16)	0.013

Abbreviations: *Height SDS* Height standard deviation scores, *BMI SDS* Body mass index standard deviation scores, *IGF-1 SDS* Insulin like growth factor-1 standard deviation scores, *TG* Triglyceride, *TC* Total cholesterol, *HDL-C* High density lipoprotein-cholesterol, *LDL-C* Low density lipoprotein cholesterol, *SUA* Serum uric acid, *BUN* Blood urea nitrogen, Cr: creatinine; *P* < 0.05 is considered to be statistically significant

### Independent association of SUA and TG levels by piecewise multivariate linear regression

**Fig. 1:**
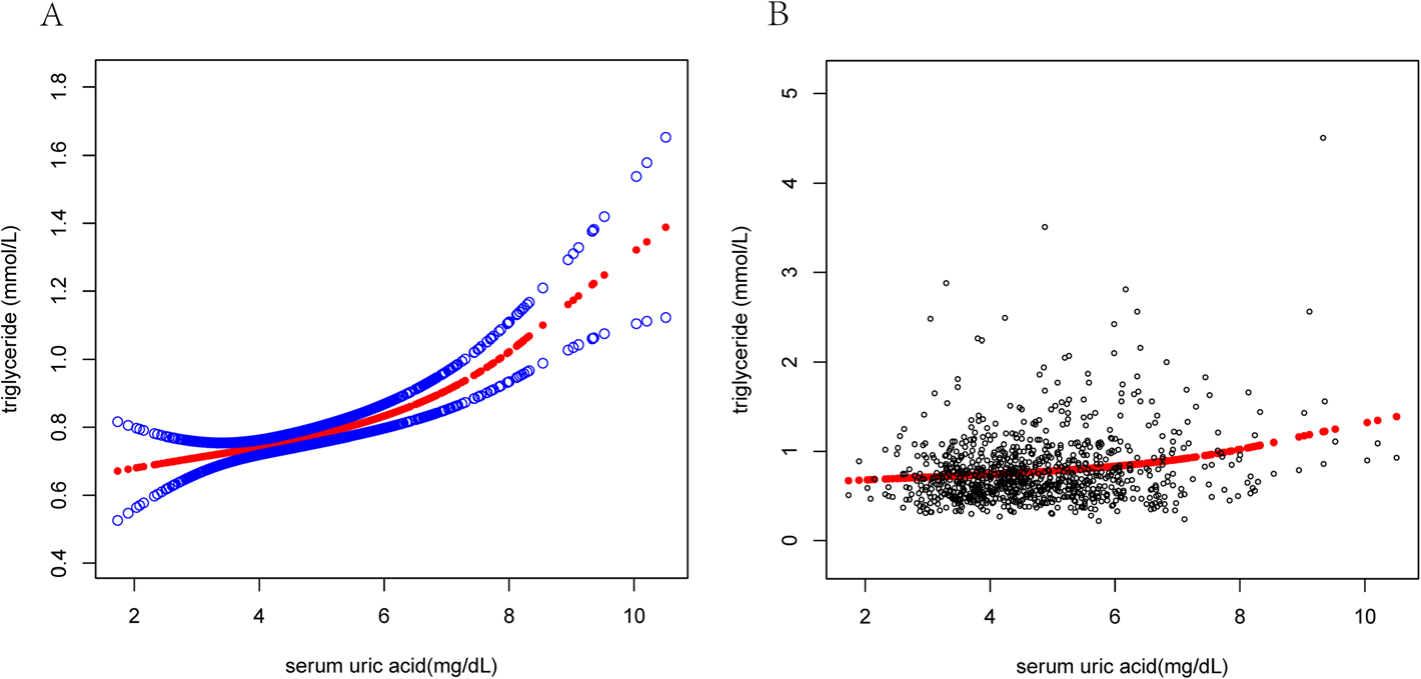
The relationship between SUA and TG levels was determined by smooth curve fitting. The curve fitting line for SUA and TG levels in children (**a**) and scatter plot of the distribution for SUA and TG levels in children (**b**) are shown. Adjusted variables: age, sex, weight, TC, pubertal stage. SUA: serum uric acid; TG: triglyceride; TC: total cholesterol

To display whether a non-linear relationship existed between the two indicators, SUA and TG, general additive models adjusted for possible confounders, including age, sex, body weight, TC and pubertal stage, were adopted. A smoothing curve indicated a non-linear correlation in the additive model and an inflection point, indicating two stages of change between SUA and TG (Fig. 1a), was found. Figure 1b is a scatter plot that displays the children’s SUA and TG levels.

Similar to the smoothing curve, as presented in Table 3, an examination of a threshold effect was performed, and the inflection point in the SUA level was 7 mg/dL. Specifically, the TG level increased as the SUA level increased when SUA levels were greater than 7 mg/dL (β = 0.13, 95% CI: 0.05–0.22; *P* = 0.002), yet no significant relationship was observed between the two variables at lower SUA concentrations (β = 0.01, 95% CI: − 0.01–0.04; *P* = 0.799). Regression models with linear and piecewise linear functions were simultaneously applied in the threshold effect analysis. The differentiation of two functions was assessed by the log-likelihood ratio test, of which a *P* value lower than 0.05 implies that a piecewise linear function was a better fit for observations; otherwise, a P value higher than 0.05 implies that the linear function was a better fit. Potential confounders that may have influenced estimating the association between SUA and TG levels were adjusted for both linear and piecewise regression models, and the results from variance inflation factor analysis showed no evidence of multicollinearity for certain confounders (Table S2).

**Table 3:** Threshold effect analysis for the relationship between SUA and TG levels

Models	TG		
	Adjusted β (95% CI)	*P* value	Adjusted R^**2**^
Model I			
One line slope	0.03 (0.01, 0.05)	< 0.001	15.26%
Model II			
Turning point	7		15.98%
< 7 slope 1	0.01 (−0.01, 0.04)	0.799	
> 7 slope 2	0.13 (0.05, 0.22)	0.002	
LRT test		0.010	

Model I, linear analysis; Model II, non-linear analysis. LRT test, Logarithmic likelihood ratio test. (*P* < 0.05 means Model II is significantly different from Model I, which indicates a non-linear relationship); Adjustment variables: age, sex, weight, TC, and pubertal stage. *SUA* Serum uric acid, *TG* Triglyceride, *TC* Total cholesterol; *P* < 0.05 is considered to be statistically significant

**Fig. 2:**
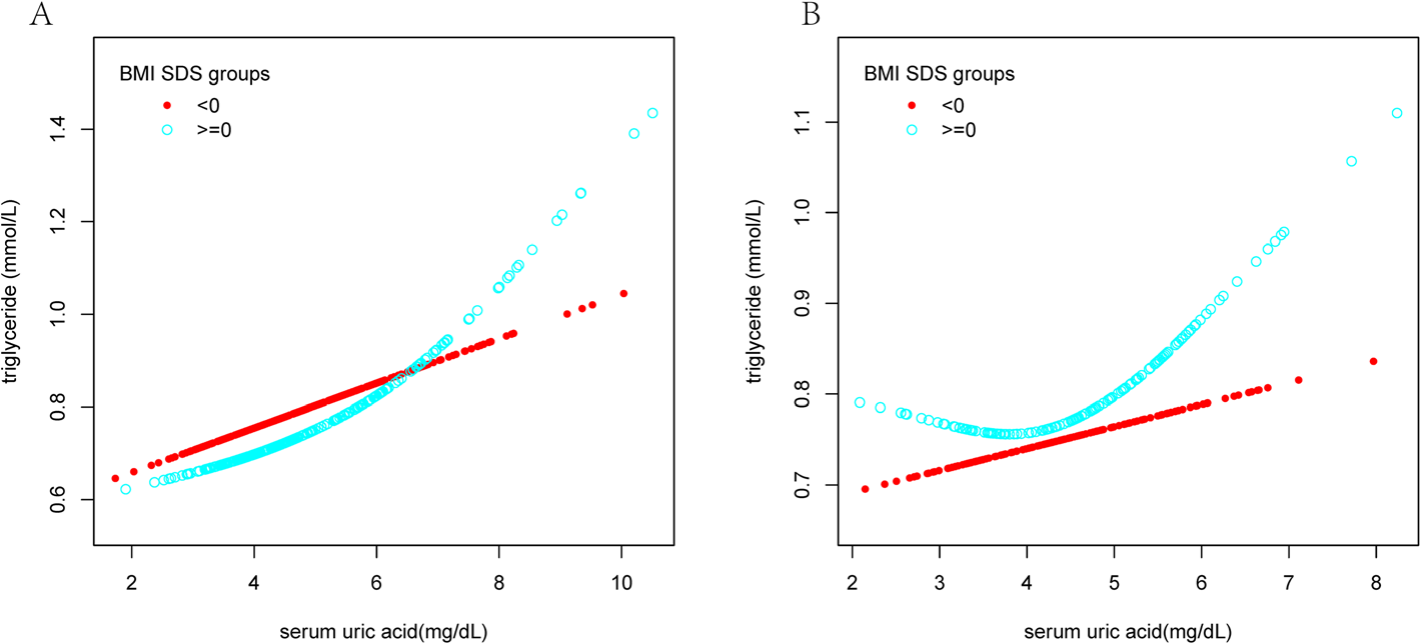
Sex-specific smooth curve fitting for the relationship between SUA and TG levels stratified by BMI SDS in males (**a**) and females (**b**). Adjusted variables: age, TC, pubertal stage. SUA: serum uric acid; TG: triglyceride; BMI SDS: body mass index standard deviation scores; TC: total cholesterol

A stratified analysis of the relationship between SUA and TG levels in different genders based on BMI was applied with a fit curve as displayed in Fig. 2 and piecewise linear regression displayed in Table 4. An important non-linear relationship between SUA and TG was observed in males and females with BMI SDS ≥ 0, and the inflection points were approximately 6.71 mg/dL and 3.93 mg/dL, respectively (Table 4). In males with BMI SDS ≥ 0, at SUA concentrations higher than 6.71 mg/dL, TG levels gradually increased with increasing SUA levels (β = 0.21, 95% CI: 0.11–0.31; *P* < 0.001), but no connection was found when SUA levels were less than the inflection point (β = 0.04, 95% CI: − 0.01–0.08; *P* = 0.088). Moreover, a similar relationship was found among females. In subjects with BMI SDS ≥ 0, TG levels increased as SUA levels increased when SUA levels were greater than 3.93 mg/dL (β = 0.10, 95% CI: 0.03–0.17; *P* = 0.005), yet no significant relationship was observed at lower SUA concentrations (β = − 0.15, 95% CI: − 0.32–0.02; *P* = 0.084). No significant associations were obtained in either sex when BMI SDS < 0.

**Table 4:** Sex-specific threshold effect analysis for the relationship between SUA and TG levels stratified by BMI SDS

Models	Male		Female	
	BMI SDS < 0	BMI SDS ≥ 0	BMI SDS < 0	BMI SDS ≥ 0
	β (95% CI) *P* value	β (95% CI) *P* value	β (95% CI) *P* value	β (95% CI) *P* value
Model I				
One line slope	0.01 (−0.02, 0.05) 0.4632	0.07 (0.04, 0.11) < 0.001	0.01 (−0.04, 0.04) 0.996	0.02 (− 0.03, 0.07) 0.515
Adjusted R^**2**^	15.85%	15.83%	4.98%	6.42%
Model II				
Turning point (K)	7.25	6.71	6.37	3.93
< K slope 1	0.03 (−0.01, 0.08) 0.948	0.04 (−0.01, 0.08) 0.088	0.02 (− 0.03, 0.07) 0.341	−0.15 (− 0.32, 0.02) 0.084
> K slope 2	0.16 (− 0.02, 0.35) 0.081	0.21 (0.11, 0.31) < 0.001	− 0.20 (− 0.53, 0.13) 0.228	0.10 (0.03, 0.17) 0.005
Adjusted R^2^	16.76%	16.70%	5.64%	7.91%
LRT test	0.204	0.007	0.196	0.019

Model I, linear analysis; Model II, non-linear analysis. LRT test, Logarithmic likelihood ratio test. (*P* < 0.05 means Model II is significantly different from Model I, which indicates a non-linear relationship); Adjustment variables: age, TC, and pubertal stage. *SUA* Serum uric acid, *TG* Triglyceride, *TC* Total cholesterol, *BMI SDS* Body mass index standard deviation scores; *P* < 0.05 is considered to be statistically significant

Results from the threshold effect analysis using the log-likelihood ratio test show that the inflection point in SUA concentration was approximately 7 mg/dL in the entire population as well as in male participants with BMI SDS ≥ 0. The inflection point was approximately 4 mg/dL in female participants with BMI SDS ≥ 0, which aligns with clinical experience that females usually have lower SUA levels than males. The adjusted R square values shown in Table 3 and Table 4 indicate the same conclusion as the log-likelihood ratio test results: in analyses of males with BMI SDS ≥ 0, females with BMI SDS ≥ 0, or the entire population, adjusted R squares were slightly greater using the piecewise function than using the linear function.

## Discussion

In the cross-sectional analysis of the cohort of children and teenagers with short stature, results showed that the amount of SUA was generally positively associated with TG concentrations. Interestingly, a non-linear association between SUA and TG levels within children having short stature was observed with an SUA threshold of 7 mg/dL. In addition, a stratified analysis was conducted, and it was found that BMI status was related to the association between SUA and TG levels. Among males and females, the relationship between SUA and TG was significant only when BMI SDS ≥ 0, showing a non-linear relationship with inflection points of 6.71 and 3.93 mg/dL, respectively. In subjects with BMI SDS < 0, the relationship between SUA and TG levels was not significant.

SUA refers to the final product of human purine metabolism. In addition to gout and kidney stones, elevated SUA levels are known as a significant risk factor for metabolic syndrome and CVD in adults [1–5]. Differences have been found in previous studies’ results on SUA and CVD [25, 26, 37]. A previous longitudinal cohort study of 127,771 adults in Taiwan identified a U-shaped association between SUA levels and CVD-related mortality rates [25]. Similarly, L. Hu et al. analysed data from 9118 US adults in the NHANES (1999–2002) and found the same relationship between SUA and CVD [26]. However, A. Dutta et al. found that SUA and CVD-related mortality were significant when the SUA level was greater than 7 mg/dL in people older than 70 years [37], which was consistent with the threshold identified in the present study. In addition, a previous study also found that high UA quartiles were associated with CVD mortality, and the association was stronger in the presence of silent myocardial infarction (SMI) [38]. Moreover, a continuous increase in hyperuricaemia in children and teenagers has been reported in recent years, and UA levels > 5.5 mg/dl were considered abnormal [39]. The relationship between SUA and TG has been inconsistent: one study reported a positive association between SUA and TG levels [28], while this relationship was not observed in another study [40]. In the present study, results showed that SUA levels were generally related to TG levels in young short stature patients, which is in line with the finding stated by Ma W et al., who conducted a study of 261 recently diagnosed Moyamoya patients in China [28]. Lurbe E et al. also found that SUA and TG levels were positively correlated, although this was a cross-sectional study in 333 obese Caucasian children aged 5 to 18 years old [21]. However, Li L et al. carried out another cross-sectional study of 409 obese Chinese adults and found no significant association between SUA and TG levels [40].

Interestingly, the relationship between SUA and TG levels was further analysed with curve fitting, and a non-linear relationship between the two was identified. TG levels increased as SUA levels increased at SUA levels higher than 7 mg/dL, whereas the relationship between SUA and TG levels was not observed at lower levels. These results show that above a certain SUA level, a strong association between SUA and TG levels exists and a simple linear evaluation might have underestimated this association. A stratified BMI analysis of the relationship between SUA and TG levels in different sexes was further performed, and it was found that there was a sex difference in the inflection point, namely, SUA levels of 6.71 mg/dL in males and 3.93 mg/dL in females, and that BMI status was related to the relationship between SUA and TG levels. A previous study reported sex-related discrepancies in the relationship between SUA and CVD levels, and the connection between SUA and CVD presented a U-shaped relationship in males and a J-shaped relationship in females [41]. S. L. Rodrigues et al. demonstrated a demand for gender-based assessments based on the association between SUA levels and cardiovascular risk factors [42]. The outcome of the present study not only found sex differences between SUA and TG levels in children and adolescents but also indicated that BMI status was related to the specific association. This result is also consistent with previous study results that reported a significant relationship between SUA and TG levels in obese children [21, 43] or displayed modelled effects of adiposity, approximated by BMI, on the relationship between CVD risk and antioxidant levels [44].

The underlying mechanism of elevated lipids caused by high levels of SUA is that SUA promotes lipid peroxidation, generates oxygen free radicals, and causes blood vessel wall inflammation. Excessive concentrations of SUA are generally considered a mediator of inflammatory endocrine disorders in adipose tissue, which may be a significant factor leading to dyslipidaemia [45]. Previous studies have confirmed the probability of SUA being an indicator of oxidative stress, inflammation development, or kidney disease [46]. Furthermore, experimental and clinical research has pointed out several possible mechanisms, including SUA levels having harmful effects on cardiovascular status, such as increased oxidative stress and nitric oxide, reduced endothelial function, promotion of inflammation, vasoconstriction and hyperplasia of vascular smooth muscle cells, insulin resistance and metabolic abnormalities [20]. In a Mendelian randomized study, SUA, as an early metabolic indicator, had an upstream effect on more traditional risk components and the development of CVD itself, suggesting that hyperuricaemia might take on a causal role in the development of CVD [47].

### Study strengths and limitations

The present study has several advantages. First, this is the first study to determine the connection between SUA and TG levels in short children and teenagers, who tend to be at higher risk of CVD in adulthood. Second, in this study, smooth curve fitting was applied to find a non-linear relationship between SUA and TG levels rather than a simple positive association. Third, a stratified analysis of the relationship between SUA and TG in different genders based on BMI was performed, and the results indicated that more importance should be placed on SUA levels in short children with higher BMI. Nevertheless, several limitations should be considered. The cross-sectional nature of the research does not permit conclusions of causal relationships. Thus, the findings of this study are suitable for Chinese children and adolescents with short stature. Discrepant outcomes may be found within other populations. Finally, the connection between low-purine food and lipid profile development in children and teenagers with short stature requires in-depth research.

## Conclusion

The present study identifies the non-linear connection between SUA and TG levels in Chinese children and adolescents with short stature. Above a certain SUA level, a powerful active connection between SUA and TG levels exists. Even though the inflection point varied in males and females, the positive relationship of the two indicators for many conditions was stable in children with high BMI. This finding suggests that the health status of short stature children and adolescents with high SUA levels should be considered, especially for those with a higher BMI, even if they do not have other conditions.

## Supplementary Information

**Additional file 1: Table S1.** Normality Distribution Test for Continuous Variables. **Table S2.** Variance Inflation Factor for the Multicollinearity Test.

## Data Availability

The datasets used and/or analysed in the current study are available from the corresponding authors upon reasonable request.

## Abbreviations

BMI SDS: Body mass index standard deviation scores
CVD: Cardiovascular disease
HDL-C: High density lipoprotein-cholesterol
Height SDS: Height standard deviation scores
IGF-1 SDS: Insulin like growth factor-1 standard deviation scores
LDL-C: Low density lipoprotein cholesterol
TC: Total cholesterol
TG: Triglyceride
SUA: Serum uric acid
