# Supplementary material for: Association between serum uric acid and triglycerides in Chinese children and adolescents with short stature
Source: Lipids Health Dis. 2021 Jan 6;20:1. doi: 10.1186/s12944-020-01429-x (PMC7786994; doi:10.1186/s12944-020-01429-x)
Supplement: Supplementary file 1 — Additional file 1: Table S1. Normality Distribution Test for Continuous Variables. Table S2. Variance Inflation Factor for the Multicollinearity Test. [file 12944_2020_1429_MOESM1_ESM.doc]

Table S1 Normality Distribution Test for Continuous Variables

|  | W-value | *P*-value for W test |
| --- | --- | --- |
| Age (years) | 0.98 | <0.0001 |
| Height (cm) | 0.96 | <0.0001 |
| Height SDS | 0.82 | <0.0001 |
| Body weight (kg) | 0.94 | <0.0001 |
| BMI (kg/m^2^) | 0.88 | <0.0001 |
| BMI SDS | 0.99 | <0.0001 |
| IGF-1 (ng/mL) | 0.93 | <0.0001 |
| IGF-1 SDS | 0.99 | <0.0001 |
| TG (mmol/L) | 0.79 | <0.0001 |
| TC (mmol/L) | 0.98 | <0.0001 |
| HDL-C (mmol/L) | 0.99 | <0.0001 |
| LDL-C (mmol/L) | 0.98 | <0.0001 |
| SUA (mg/dL) | 0.96 | <0.0001 |
| Cr (μmol/L) | 0.32 | <0.0001 |
| BUN (mmol/L) | 0.23 | <0.0001 |

Abbreviations: Height SDS: height standard deviation scores; BMI SDS: body mass index standard deviation scores; IGF-1 SDS: insulin like growth factor-1 standard deviation scores; TG: triglyceride; TC: total cholesterol; HDL-C: high density lipoprotein-cholesterol; LDL-C: low density lipoprotein cholesterol; SUA: serum uric acid; BUN: blood urea nitrogen, Cr: creatinine; W test: Shapiro-Wilk distribution test; W-value: statistic of Shapiro-Wilk test; *P* < 0.05 is considered to be statistically significant.

Table S2 Variance Inflation Factor for the Multicollinearity Test

| Variables | VIF |
| --- | --- |
| TG (mmol/L) | 1.1 |
| SUA (mg/dL) | 1.3 |
| Age (years) | 2.1 |
| Sex | 1.2 |
| Pubertal stage | 1.8 |
| TC (mmol/L) | 1 |
| Body weight (kg) | 2.8 |

Abbreviations: TG: triglyceride; SUA: serum uric acid; TC: total cholesterol; VIF: variance inflation factor that is considered > 5 to indicate existence of multicollinearity in certain variable.
